# Identification and validation of dysregulated MAPK7 (ERK5) as a novel oncogenic target in squamous cell lung and esophageal carcinoma

**DOI:** 10.1186/s12885-015-1455-y

**Published:** 2015-06-04

**Authors:** Paul R. Gavine, Mei Wang, Dehua Yu, Eva Hu, Chunlei Huang, Jenny Xia, Xinying Su, Joan Fan, Tianwei Zhang, Qingqing Ye, Li Zheng, Guanshan Zhu, Ziliang Qian, Qingquan Luo, Ying Yong Hou, Qunsheng Ji

**Affiliations:** 1 Innovation Center China, AstraZeneca Global R&D, Zhangjiang Hi-Tech Park, Shanghai, 201203 People’s Republic of China; 2 Shanghai Chest Hospital, Shanghai, People’s Republic of China; 3 Shanghai Zhongshan Hospital, Shanghai, People’s Republic of China

**Keywords:** MAPK7, ERK5, Oncogene, Kinase, Inhibitor

## Abstract

**Background:**

MAPK7/ERK5 (extracellular-signal-regulated kinase 5) functions within a canonical three-tiered MAPK (mitogen activated protein kinase) signaling cascade comprising MEK (MAPK/ERK kinase) 5, MEKK(MEK kinase) 2/3 and ERK5 itself. Despite being the least well studied of the MAPK-modules, evidence supports a role for MAPK7-signaling in the pathology of several cancer types.

**Methods and results:**

Fluorescence *in situ* hybridization (FISH) analysis identified MAPK7 gene amplification in 4 % (3/74) of non-small cell lung cancers (NSCLC) (enriched to 6 % (3/49) in squamous cell carcinoma) and 2 % (2/95) of squamous esophageal cancers (sqEC). Immunohistochemical (IHC) analysis revealed a good correlation between MAPK7 gene amplification and protein expression. MAPK7 was validated as a proliferative oncogenic driver by performing *in vitro* siRNA knockdown of *MAPK7* in tumor cell lines. Finally, a novel MEK5/MAPK7 co-transfected HEK293 cell line was developed and used for routine cell-based pharmacodynamic screening. Phosphorylation antibody microarray analysis also identified novel downstream pharmacodynamic (PD) biomarkers of MAPK7 kinase inhibition in tumor cells (pMEF2A and pMEF2D).

**Conclusions:**

Together, these data highlight a broader role for dysregulated MAPK7 in driving tumorigenesis within niche populations of highly prevalent tumor types, and describe current efforts in establishing a robust drug discovery screening cascade.

**Electronic supplementary material:**

The online version of this article (doi:10.1186/s12885-015-1455-y) contains supplementary material, which is available to authorized users.

## Background

Mitogen activated protein (MAP) kinase signaling pathways are highly evolutionarily conserved throughout eukaryotes and represent a key mechanism in the transduction of intracellular signals. Of the four MAP kinases which exist in mammalian cells (ERK1/2, JNK, p38 and ERK5 [1–3], ERK (extracellular-signal-regulated kinase) 5 is the least well studied and most structurally divergent of the family. The ERK5 protein, encoded by the *MAPK7* gene [4], contains an N-terminal kinase domain and a large C-terminal segment, containing a transactivation domain and nuclear localization and export sequences (NLS/NES). ERK5 is the effector kinase of a canonical kinase module containing; MEK (MAPK/ERK kinase) 5, MEKK (MEK kinase) 2/3 and ERK5 itself [5].

Under normal physiological conditions, MEK5 and ERK5 are activated by growth factors and cellular stresses [6, 7] and, through the use of embryonic gene knockouts of *MEK5* or *MAPK7*, have been shown to contribute largely to blood vessel and cardiac formation during development [8, 9]. *In vitro* muscle differentiation systems have highlighted prominent roles for ERK5 signaling in muscle development [10], whilst in adult tissues, the pathway plays a role in regulating the proliferation and survival of endothelial cells and various immune-derived cell populations [11–14].

In the context of cancer, clinical evidence suggests a role for dysregulated MEK5/ERK5 signaling as a driver of tumorigenesis in several cancers. Specifically in breast cancer, increased ERK5 protein levels are associated with decreased disease-free survival and furthermore, MEK5 expression is up-regulated by constitutive activation of STAT (signal transducer and activator of transcription) 3, commonly detected in advanced breast cancer [15, 16]. The ERK5 pathway also appears to play a role in mediating chemoresistance in breast cancer cells and contributes to neuregulin signaling in breast cancer cells overexpressing ErbB2 [17, 18]. In prostate cancer, MEK5 is overexpressed and is associated with bone metastases, invasive potential and corresponding poor survival [19]. Furthermore, in hepatocellular carcinoma (HCC), genetic dysregulation of *MAPK7* expression through amplification of 17p11 is detectable in around 50 % of primary HCC tumors [20]. In the same study, preclinical validation work using small-interfering RNA (siRNA) suppression of *MAPK7* expression in amplified cell lines confirmed a role for dysregulated MAPK7 in controlling mitotic entry.

In the work reported here, we identified genetic dysregulation of *MAPK7* and protein overexpression in clinical samples of non-small cell lung cancer (NSCLC) and esophageal cancer (EC) of Asian origin, using array comparative genomic hybridization (aCGH) and FISH (fluorescent *in-situ* hybridization) technologies. Importantly, by suppressing expression within *MAPK7* amplified cell lines, we were able to validate MAPK7 as a driver of tumor cell proliferation and engineer a stable cell line assay for screening of candidate MAPK7 small molecule kinase inhibitors. Lastly, using reverse-phase protein chip arrays, our work identified potential pharmacodynamic biomarkers of MAPK7 kinase inhibition within *MAPK7*-amplified tumor cell lines. In summary, the work here identifies and validates a novel role for dysregulated *MAPK7* as a tumor driver in clinical samples of NSCLC and EC, and outlines aspects of preliminary work in developing a drug discovery programme to identify novel small molecule inhibitors of MAPK7 kinase activity.

## Results

### Identification of dysregulated MAPK7 expression in Chinese squamous cell lung and esophageal carcinoma patient samples

**Fig. 1:**
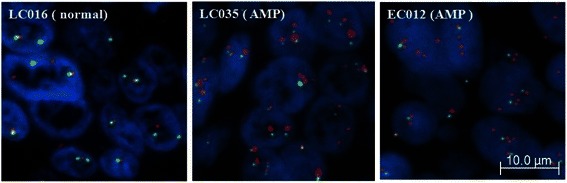
Representative FISH data showing *MAPK7* gene amplification in NSCLC and sqEC tumor tissues. FISH probes were generated for *MAPK7* (red) and internal *CEP17* control (green) genes. *MAPK7* non-amplified tumor tissue is represented by image LC016, while *MAPK7* amplified lung and esophageal tumor tissues are shown in images LC035 and EC012, respectively. Gene amplification criteria were defined as a *MAPK7/CEP17* ratio of >2 across at least tumor 50 cells

**Fig. 2:**
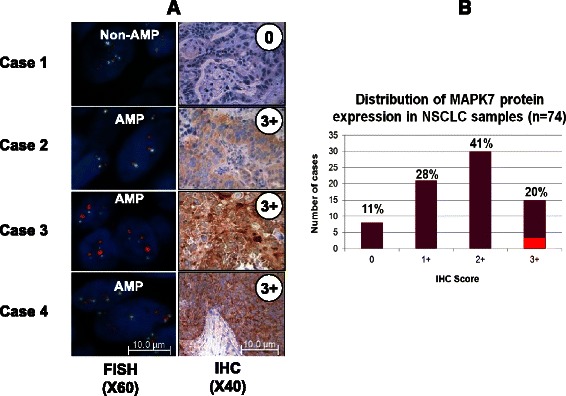
MAPK7 gene amplification correlates with high level protein expression. **a** Representative FISH and IHC images of MAPK7 expression from 4 cases of NSCLC (3 *MAPK7* amplified and 1 non-amplified). **b** Histogram view of the distribution of MAPK7 IHC scores across the cohort of 74 NSCLC tumor samples. The three *MAPK7* amplified samples are shown in red within the IHC 3+ category

To explore MAPK7 tumor expression profiles in Asian cancer patients, we collected 74 non-small cell lung cancers and 95 squamous esophageal cancers of Chinese origin. Fluorescent *in situ* hybridization (FISH) analysis identified high level *MAPK7* gene amplification in 4 % (3/74) of NSCLC (enriched to 6 % (3/49) in squamous cell carcinoma) and 2 % (2/95) of sqEC (Fig. 1 and Table  1). In order to investigate correlations between genetic dysregulation of *MAPK7* expression and corresponding protein expression, immunohistochemical (IHC) analysis of the same NSCLC tissue samples was performed. Analysis revealed that all 3 *MAPK7* amplified cases had corresponding high level MAPK7 protein expression (defined as IHC3+), suggesting a good correlation of *MAPK7* gene amplification with high level protein expression (Fig. 2A and Additional file 1: Figure S1). Importantly however, this analysis also identified a high prevalence of MAPK7 protein expression in the absence of gene amplification, with 20 % of samples (15/74) staining strongly (IHC3+) for MAPK7 protein expression (Fig. 2B). Of the remaining samples, 41 % (30/74), 28 % (21/74) and 11 % (8/74) stained IHC2+, IHC1+ and IHC0 for MAPK7 protein, respectively.

**Table 1:** MAPK7 gene amplification prevalence in Chinese NSCLC and sqEC

Tumor types	MAPK7 AMP prevalence
NSCLC	4 % (3/74) (enriched to 3/49 in squamous cell lung)
sqEC	2 % (2/95)

### MAPK7 is a driver of tumor cell proliferation in dysregulated cell lines

**Fig. 3:**
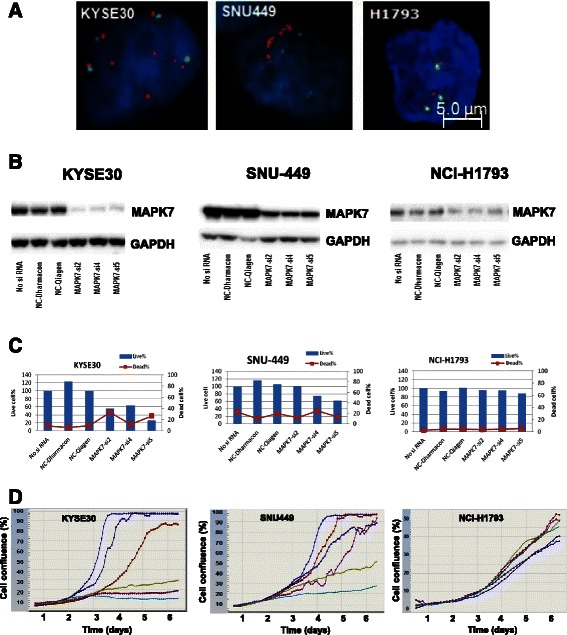
*MAPK7* amplification correlates with protein expression and drives proliferation in tumor cell lines KYSE30 and SNU449. **a** Representative FISH images of the *MAPK7* amplified cell lines KYSE30 and SNU449, and the MAPK7 diploid line, H1793. FISH probes were generated for *MAPK7* (red) and internal *CEP17* control (green) genes. **b** Western blot MAPK7 protein expression analysis of cell lines following 4 day treatment with siRNA controls (left 3 lanes in each panel) or *MAPK7*-directed siRNAs (right 3 lanes in each panel). GAPDH is included as a protein loading control. **c** 4 days post-siRNA transfection, tumor cell lines were assessed for cell survival using an Acumen platform and utilizing ‘live/dead’ cell stains (for details see ‘Methods’). **d** Following siRNA transfection, cell growth dynamics were captured over a 6 day period in all treatment groups using an Incucyte cell confluence imaging platform. Treatment groups are represented by colored lines as follows: Light blue – No SiRNA, Dark blue – NC Dharmacon, Red – NC Qiagen, Purple – MAPK7si2, Gold – MAPK7si4, Turquoise – MAPK7si5

In order to test the hypothesis that dysregulation of MAPK7 signaling could drive tumor cell proliferation, we undertook a number of studies to explore the functional consequences of silencing *MAPK7* gene expression in MAPK7 dysregulated tumor cell lines. Two *MAPK7* gene amplified and overexpressing cell lines (KYSE30 and SNU449) and a MAPK7 ‘normal’ cell line (NCI-H1793), were selected for study. Detailed *MAPK7* gene copy number and *MAPK7/CEP17* ratios for these cell lines are tabulated in Additional File 2: Table S1. FISH staining of cell pellets confirmed *MAPK7* gene amplification in cell lines KYSE30 and SNU449, but not in NCI-H1793 (Fig. 3a), and Western blotting of lysates confirmed higher level MAPK7 protein expression in KYSE30 and SNU449, but lower levels in NCI-H1793 (Fig. 3b – control lanes and Additional file 3: Figure S2). Next, we performed *in vitro* transfection of these cell lines using *MAPK7* small interfering RNA (siRNA) to silence MAPK7 gene expression. Cell lines were transfected over a 4-day period and cell lysates were taken for Western blot analysis of MAPK7 protein expression (Fig. 3b). In the MAPK7 dysregulated cell lines KYSE30 and SNU449, MAPK7 protein expression was reduced by 90 and 70 % respectively, using three separate MAPK7 siRNA constructs. In the NCI-H1793 control cell line, despite low baseline MAPK7 protein expression, significant expression knockdown of around 60 % was also achieved. In parallel, and to evaluate the impact of *MAPK7* gene silencing on cell proliferation, identical transfection groups were assayed using an Acumen-based ‘live/dead’ cell enumeration assay over a 6 day period. In the KYSE30 cell line, near complete knockdown of MAPK7 protein expression using all three of the MAPK7 siRNA constructs led to significant reductions in cell proliferation in each case, accompanied by elevations in the numbers of dead cells to between 20 and 40 % after 6 days. In the SNU449 cell line, partial MAPK7 knockdown resulted in significant reductions in the number of live cells after 6 days using 2 of the 3 siRNA constructs, but in this case, with no appreciable change in dead cell counts. Within the NCI-H1793 control cell line, knockdown of MAPK7 protein expression to around 60 % of control levels had no effect on either cell proliferation or cell death. To further confirm these findings, we measured the dynamics of cell line growth over a 6 day period using an Incucyte cell imaging platform. Consistent with the ‘live/dead’ cell assay, MAPK7 knockdown greatly reduced cell proliferation in KYS30 and SNU449 cell lines, but had no effect on NCI-H1793 (Fig. 3d). Two days post-transfection, MAPK7 siRNA-transfected tumor cells failed to follow the same exponential growth dynamics of the control and scrambled siRNA treatment groups, instead displaying suppressed cell growth, and in the case of KYSE30, reductions in the degree of cell confluence consistent with the increased cell death observed in the ‘live/dead’cell assay.

### Development of a MEK5A/MAPK7 cell-based *in vitro* pharmacodynamic ELISA co-expression assay for small molecule drug screening

**Fig. 4:**
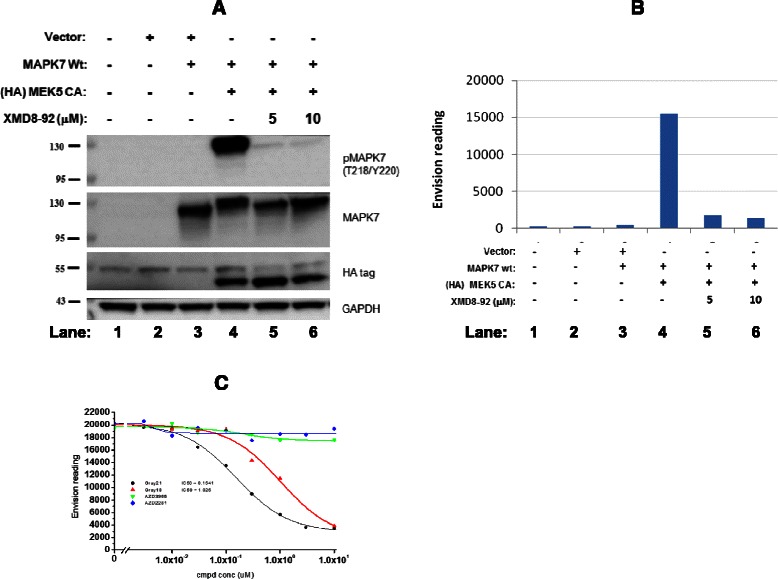
Development and validation of a MEK5A/MAPK7 cell-based *in vitro* pharmacodynamic ELISA co-expression assay. **a** Western blot analysis of HEK293 cell transfectants. HEK293 cells were transfected with plasmid vectors and/or treated with compound as indicated in the matrix (details in ‘Methods’ section). Cell lysates were prepared and analysed using Western blot to detect phospho-MAPK7 (T218/Y220), total MAPK7, HA-tag and GAPDH. **b** Using the same cellular lysates, a quantitative sandwich ELISA assay was developed by coating ELISA plates with a total MAPK7 capture antibody, incubating with lysate and then using a phospho-MAPK7 (T218/Y220) detection antibody. Phospho-MAPK7 signals were then quantified using an envision reader. **c** Further validation of the pMAPK7 ELISA assay was performed by running dose–response experiments with 4 small molecules; 2 MAPK7 inhibitors and 2 unrelated kinase inhibitors (AZD2281 and AZD3965)

To facilitate drug discovery efforts to target the MAPK7-signaling axis, we next sought to develop a cell-based pharmacodynamic assay to enable the effective screening of compounds possessing MAPK7 kinase inhibitory activity. Although previous reports have documented the ability of exogenously added epidermal growth factor (EGF) to induce direct cellular activation of MAPK7, this effect is only reliably detectable using a non-quantitative gel-shift assay [21]. Using a more artificial *in vitro* system, Kato *et al.* co-expressed a constitutively active form of MEK5 (MEK5CA) with MAPK7 in CHO-K1 cells and demonstrated the kinase specificity of MEK5 via direct phosphorylation and activation of MAPK7 [22]. Taking a similar approach to ensure robust and specific activation of MAPK7, we generated a stable co-expression system utilizing MEK5CA and MAPK7 within a HEK293 cell background. Western blot analysis demonstrated clear induction of MAPK7 Thr218/Tyr220 phosphorylation by MEK5CA, and importantly, this phosphorylation could be blocked using the commercially available and specific MAPK7 tool compound, XMD8-92 (Fig. 4a) [21]. This conclusion was further supported by evidence of a ‘gel shift’ in the molecular weight of MAPK7 upon co-expression with MEK5CA, indicative of post-translational modifications (likely phosphorylation). Moreover, upon pre-treatment with XMD8-92, this gel-shift did not occur and MAPK7 remained in a lower molecular weight form. Having achieved a significant and dynamic phospho-MAPK7 signal detectable by Western blot, we next sought to develop a higher throughput ELISA-based phospho-MAPK7 assay. Cell lysates from co-expression studies were tested and optimized in a custom ‘sandwich ELISA’ assay using commercially available antibodies pairs (described in ‘Methods’ section), with the results shown in Fig. 4b. High level expression of phosphorylated MAPK7 protein was detectable using the optimized assay platform (lane 4), with an acceptable signal:noise ratio observed upon analysis of XMD8-92 treated cell lysates (lanes 5 and 6). Further validation of this assay was performed using 2 novel small molecules, Gray#18 and Gray#21 (confirmed as MAPK7 kinase inhibitors using a biochemical kinase assay) and 2 unrelated kinase inhibitors, AZD2281 (a PARP inhibitor) and AZD3965 (an MCT1 inhibitor). MAPK7 half-maximal cellular inhibitory concentrations (IC_50_) were determined for all four agents (Fig. 4c).

### Identification of downstream biomarkers of MAPK7 activity

**Fig. 5:**
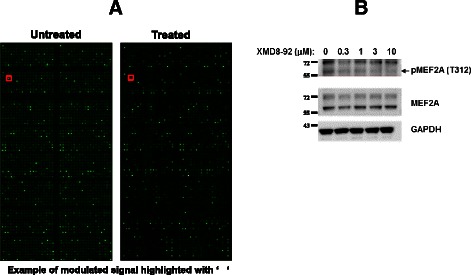
Phosphorylation antibody microarray analysis of KYSE30 cells treated with XMD8-92 and confirmation of pMEF2A modulation. **a** KYSE30 cells were treated (or not) for 2 h with 10 μM XMD8-92 and cell lysates were then prepared and analysed using phospho-antibody microarray. Actual microarray visualizations are presented. **b** KYSE30 cells were treated for 2 h with a range of XMD8-92 concentrations and cell lysates prepared for Western blotting using antibodies to detect pMEF2A (T312), total MEF2A and GAPDH

To enable more comprehensive characterisation of MAPK7-pathway signaling, we sought to identify cellular pharmacodynamic biomarkers using a reverse-phase protein array (RPPA) platform. Protein extracts were prepared from KYSE30 cells, treated (and untreated) for 2 h with 10 μM XMD8-92, and then applied to the antibody microarray. Quality control and data normalization was performed using a standardized and validated antibody set and actual array chip images are shown in Fig. 5a. Following automated array chip analysis, of 1,318 antibodies (details contained in ‘Methods’ section) we identified 5 showing significant signal modulation upon treatment with XMD8-92. Compared to untreated control cells, pCDC25C (S216), pCDKN1 (T145), pMEF2A (S408) and pMEF2D (S444) all showed significant reductions with ‘% of control’ values of 39.8, 41.9, 44.7 and 45.1, respectively (Table 2). To corroborate these findings, direct pMEF2A modulation by MAPK7 inhibition was confirmed by immunoblot analysis using an antibody raised against phosphorylated Thr312 of MEF2A (Fig. 5b).

**Table 2:** Protein targets identified from phosphorylation antibody microarray

Protein target	Signal	SEM (%)
	(% of control)	
CDC25C (p-S216)	39.80 %	2
CDKN1 (p-T145)	41.90 %	3.9
MEF2A (p-S408)	44.70 %	1.9
MEF2D (p-S444)	45.10 %	0.5
MEF2A (p-T312)	54.50 %	7.4

## Discussion

Dysregulated MAPK7 signaling has been demonstrated to play key roles in uncontrolled cell proliferation across several tumor types [15, 17, 19]. The data herein provide a first description of the identification of clinical squamous cell lung and esophageal tumor samples harbouring gene amplified and overexpressed MAPK7. Intriguingly, in addition to the 3 clinical cases showing a correlation between *MAPK7* amplification and high level protein expression (IHC3+), we also identified a further 12 cases with IHC3+ protein staining in the absence of *MAPK7* amplification, raising the question of whether *MAPK7* gene amplification, protein overexpression, or both, are required for driving tumor proliferation. Despite our cell lines showing a good correlation between *MAPK7* gene copy number/ratio and protein expression (Additional file 2: Table S1 and Fig. 3b), we were unable to fully answer this question with the cell lines and samples available. However, this remains a key question and is a focus of ongoing efforts in a research environment where the development of companion diagnostics for molecularly targeted therapeutic agents is increasingly required to ensure accurate selection of those patients most likely to benefit. Albeit within a relatively small number of lung cancer samples, gene amplification and overexpression of MAPK7 appeared to be a distinct molecular feature, as of the 3 samples, only 1 contained an *EGFR L858R* mutation and none had *KRas* mutation (data not shown). Although our data demonstrate coordinate MAPK7 gene amplification and protein overexpression, it should be noted that previous studies have identified additional mechanisms through which MAPK7 activity can be regulated [23, 24]. Detailed exploration of the precise molecular mechanisms of MAPK7 dysregulation was outwith the scope of our work here, however our data do suggest that dysregulated MAPK7 may provide a further level of disease segmentation within squamous cell lung cancer, which crucially, is a disease with high unmet need and currently has no approved targeted therapeutics [25].

Targeted siRNA knockdown of *MAPK7* in two dysregulated cell lines confirmed a driving role for MAPK7 in tumor cell proliferation *in vitro*. Interestingly, although a good correlation was observed between *MAPK7* gene amplification and protein expression in both cell lines, targeted knockdown had slightly different functional effects. Partial knockdown of MAPK7 expression in the SNU449 cell line resulted in anti-proliferative activity but no appreciable increase in cell death above control levels. In contrast, near-complete suppression of MAPK7 expression in the KYSE30 cell line resulted in anti-proliferative activity and significant increases in cell death above background levels. It is currently unclear whether this difference was attributable to variation in the degree of siRNA *MAPK7* suppression, or perhaps due to the cellular background and pathway ‘wiring’. Importantly however, significant suppression of *MAPK7* expression and a lack of any effect on cell proliferation or cell death in the non-dysregulated MAPK7 cell line, NCI-H1793, confirmed a role for dysregulated MAPK7 in driving uncontrolled cell proliferation. The relatively benign effect of MAPK7 suppression in the MAPK7 ‘normal’ NCI-H1793 cell line is consistent with the results of previous inducible genetic *MAPK7* knockout studies in adult mice, which despite perturbing vascular integrity, had no wider effects on the whole animal [11].

To facilitate validation of our cell-based MAPK7 pharmacodynamic ELISA assay and provide a positive control in our biomarker screen, XMD8-92 served as an excellent tool compound due to its highly selective nature. Profiling of this agent against a panel of 402 diverse kinases identified MAPK7 as the most potently inhibited kinase (dissociation constant, K_D_ = 80nM), followed by DCAMKL2 (K_D_ = 190nM), TNK1 (K_D_ = 890nM) and PLK4 (K_D_ = 600nM) [21]. Notably, none of these three kinases are known to have any associations with MAPK7 biology and indeed, perform roles in cellular processes highly distinct to that of MAPK7. Interestingly, and in contrast to the *MAPK7* gene knockout work described above, *in vivo* studies using XMD8-92 did not lead to any vascular abnormalities, supporting the concept that targeting MAPK7 kinase activity (as opposed to whole gene knockout) may be a viable therapeutic strategy. Our identification of MEF2A and MEF2D (‘myocyte enhancer factors’) as novel pharmacodynamic biomarkers of MAPK7 inhibition is notable. These transcription factors bind and activate the transcription of numerous muscle-specific, growth-factor and stress controlled genes involved primarily in (but not limited to) skeletal and cardiac muscle development. Interestingly however, oncogenic activity has been attributed to MEF2D fusion proteins in acute lymphoblastic leukaemia [26], underlining a broader role for MAPK7 signaling in oncogenesis. Modulation of CDC25C (pSer216) and CDKN1 (pThr145) indirectly by XMD8-92 treatment are consistent with previously suggested roles for dysregulated MAPK7 in controlling mitotic entry [20]. Although we were able to confirm modulation of MEF2A (pThr312) by XMD8-92 using immunoblot analysis, direct confirmation of the additional biomarkers was not possible due to a lack of high quality antibodies. Efforts are currently ongoing to generate antibody reagents and perform further evaluation.

## Conclusions

Taken together, our data provide the first reported incidences of dysregulated *MAPK7* expression in clinical samples of squamous cell lung and esophageal carcinoma. Using *MAPK7* siRNA, we validate a role for dysregulated *MAPK7* in driving cell proliferation in established tumor cell lines and describe the development of a cell based ELISA assay to support screening of novel MAPK7 kinase inhibitors. Finally, we highlight the identification of several putative novel pharmacodynamic biomarkers of cellular MAPK7 signaling. This data affirms MAPK7 as an attractive therapeutic oncology target and outlines aspects of preliminary work in developing a drug discovery program to identify novel small molecule inhibitors of MAPK7 kinase activity.

## Methods

### Clinical samples and cell lines

74 NSCLC and 95 sqEC cancer clinical samples were collected from Beijing tumor hospital (Beijing, China) with signed patient informed consent. This study was approved by the ethics committee of the Beijing tumor hospital. Cell lines were obtained from commercial sources (American Type Culture Collection, Japan Health Science Research Resource Bank) or from AstraZeneca internal cell banks.

### FISH detection

The MAPK7 FISH probe was generated internally by directly labeling BAC (CTD-2387H16) DNA with Spectrum Red (Vysis, Cat # 30–803400). The CEP17-Spectrum Green probe (Vysis, 32–132017) for the centromeric region of chromosome 17 was used as internal control.

FISH assays were performed on 4 μm dewaxed and dehydrated FFPE sections. The SpotLight Tissue pretreatment Kit (Invitrogen, 00–8401) was used for pretreatment (boiled in reagent 1 for ~15 min then coated with reagent 2 for ~10 min, minor time adjustments were made for individual samples). Sections and probes were codenaturated at 80 °C for 5 min and then hybridized at 37 °C for 48 h. After a quick post wash off process (0.3 % NP40/1 × SSC at 75.5 °C for 5 min, twice in 2 × SSC at room temperature for 2 min), sections were finally mounted with 0.3 μg/ml DAPI (Vector, H-1200), and stored at 4 °C avoiding light for at least 30 min prior to scoring.

### siRNA transfection

In vitro siRNA transfection was performed using HiPerFect transfection reagent (Qiagen) following the manufacturer’s protocol. Briefly, cells (1 × 10^4^/well in 96-well plate or 2 × 10^5^/well in 6-well plate) were transfected with 40 nM of MAPK7 siRNAs (Sigma) or non-silencing siRNAs (Dharmacon) as control. The confluence of cells reflecting cell growth was monitored by Incucyte (Essen) over time after siRNA treatment. At 96 h post transfection, the cells were staining with final concentration of 1.5 μM of propidium iodide (Invitrogen, P3566) and 10 μM of Hoechst (Invitrogen, H21486), and the survival rate of cells was detected by Acumen X3 (TTP LabTech).

### Western blotting

Ninety-six h post siRNA transfection, cell lysates were collected and subjected to SDS/PAGE and transferred on to PVDF membrane. The membrane was blocked with 5 % skimmed milk in TBS-Tween20 overnight at 4 °C, before being incubated with primary antibody for 2 h at room temperature or overnight at 4 °C. After washing, the membrane was incubated with HRP (horseradish peroxidase)-conjugated secondary antibody for 1 h at room temperature. Signals were visualized using Amersham ECL detection reagents (GE health). The primary antibodies included: MAPK7 antibody (CST, #3552), pMAPK7 (T218/Y220) (CST, #3371), pMEF2A (T312) (Abcam, ab#30644), MEF2A (Abcam, ab#32866), and HA (Roche, #11 583 816 001).

### Generation of HEK293 cells coexpressing MEK5CA and MAPK7

The constitutively active MEK5 (MEK5CA) and MAPK7 plasmids were constructed as published [22]. HEK293 cells were seeded at 5,000,000/100 ml/T150 flask overnight and then co-transfected with 10 μg MEK5CA and 10 μg MAPK7 by using 60 μl of Roche X-treme Gene 9 DNA transfection reagent (Roche, #6365779001) for 48 h. Cells were then harvested and frozen down in aliquots.

### Cell-based pMAPK7 ELISA surrogate PD assay

Co-transfected HEK293 cells (described above) were aliquotted and seeded at a density of 10,000 cells/100 μl/96-well for 4 h, and then treated with various concentrations of XMD8-92 for 2 h at 37 °C in a cell incubator (Thermo). After washing out cell culture medium, 110 μl/well of RIPA-cocktail buffer was added to lyse the cells overnight. On the same day, 96-well NUNC plates (PerkinElmer, AAAND-0001) were coated with 100 μl/well of MAPK7 capture antibody (R&D, AF2848) at 1 μg/ml at 4 °C overnight. Next day, plates were then washed with buffer (PBS containing 0.05 % Tween20) 3 times, and blocked with 2 % BSA-wash buffer for 1 h. Cell lysates were then added (100 μl/well) for 2 h, followed by washing 3 times. pMAPK7 (T218/Y220) detection antibody (Santa Cruz, sc-135761) was added at 5 μg/ml for 2 h, and plates then washed before incubating with secondary antibody (PerkinElmer, AD0207) for 1 h. Plates were given a final wash, enhancement solution added (PerkinElmer, 1244-104) for 30 min, before reading on the Envision reader(PerkinElmer).

### Phosphorylation antibody microarray

The phosphorylation explorer antibody microarray chip PEX100, containing 1,318 well-characterized site-specific antibodies, was purchased from Full Moon Biosystems (Sunnyvale, CA, USA). Details of these antibodies can be accessed via the vendors website (http://www.fullmoonbio.com/product/phospho-explorer-antibody-array/). KYSE30 cells (2 × 10^7^ cells per 10-cm plate) were treated with or without 10 μM XMD8-92 for 2 h. Cells were washed with cold 1X PBS (4 °C) and collected by scraping from the plate. Cells were spun briefly in a microcentrifuge to remove culture media from the cells. Three additional washes were performed using cold 1X PBS (4 °C). Cells were centrifuged again at 4 °C and supernatant discarded. Cell pellets were shipped to the vendor and antibody microarray’s run according to the manufacturer’s instruction. Duplicate chips were run for both control and treated lysates groups and ‘% of control’ values calculated according to normalised average plate readings.

## Additional files

Additional file 1: Figure S1. Representative FISH and IHC staining images of NSCLC tissue samples. Circled numbers within the IHC images refer to ‘IHC 1+’ and ‘IHC 2+’ staining intensities.
Additional file 2: Table S1. Detailed cell line MAPK7 FISH data.
Additional file 3: Figure S2. Western blot analysis of tumor cell lines for Erk5 protein. Cell lysates were prepared and analysed for detection of total Erk5 and GAPDH as described in ‘materials and methods’.

## References

[1] Raman M, Chen W, Cobb MH (2007). Differential regulation and properties of MAPKs. Oncogene.

[2] Chang L, Karin M (2001). Mammalian MAP kinase signalling cascades. Nature.

[3] Pearson G, Robinson F, Beers Gibson T, Xu BE, Karandikar M, Berman K (2001). Mitogen-activated protein (MAP) kinase pathways: regulation and physiological functions. Endocr Rev.

[4] Zhou G, Bao ZQ, Dixon JE (1995). Components of a new human protein kinase signal transduction pathway. J Biol Chem.

[5] Lee JD, Ulevitch RJ, Han J (1995). Primary structure of BMK1: a new mammalian map kinase. Biochem Biophys Res Commun.

[6] Abe J, Kusuhara M, Ulevitch RJ, Berk BC, Lee JD (1996). Big mitogen-activated protein kinase 1 (BMK1) is a redox-sensitive kinase. J Biol Chem.

[7] Kamakura S, Moriguchi T, Nishida E (1999). Activation of the protein kinase ERK5/BMK1 by receptor tyrosine kinases. Identification and characterization of a signaling pathway to the nucleus. J Biol Chem.

[8] Wang X, Merritt AJ, Seyfried J, Guo C, Papadakis ES, Finegan KG (2005). Targeted deletion of mek5 causes early embryonic death and defects in the extracellular signal-regulated kinase 5/myocyte enhancer factor 2 cell survival pathway. Mol Cell Biol.

[9] Regan CP, Li W, Boucher DM, Spatz S, Su MS, Kuida K (2002). Erk5 null mice display multiple extraembryonic vascular and embryonic cardiovascular defects. Proc Natl Acad Sci U S A.

[10] Dinev D, Jordan BW, Neufeld B, Lee JD, Lindemann D, Rapp UR (2001). Extracellular signal regulated kinase 5 (ERK5) is required for the differentiation of muscle cells. EMBO Rep.

[11] Hayashi M, Kim SW, Imanaka-Yoshida K, Yoshida T, Abel ED, Eliceiri B (2004). Targeted deletion of BMK1/ERK5 in adult mice perturbs vascular integrity and leads to endothelial failure. J Clin Invest.

[12] Sohn SJ, Lewis GM, Winoto A (2008). Non-redundant function of the MEK5-ERK5 pathway in thymocyte apoptosis. EMBO J.

[13] Rovida E, Spinelli E, Sdelci S, Barbetti V, Morandi A, Giuntoli S (2008). ERK5/BMK1 is indispensable for optimal colony-stimulating factor 1 (CSF-1)-induced proliferation in macrophages in a Src-dependent fashion. Journal of immunology (Baltimore, Md: 1950).

[14] Garaude J, Cherni S, Kaminski S, Delepine E, Chable-Bessia C, Benkirane M (2006). ERK5 activates NF-kappaB in leukemic T cells and is essential for their growth in vivo. Journal of immunology (Baltimore, Md: 1950).

[15] Song H, Jin X, Lin J (2004). Stat3 upregulates MEK5 expression in human breast cancer cells. Oncogene.

[16] Montero JC, Ocana A, Abad M, Ortiz-Ruiz MJ, Pandiella A, Esparis-Ogando A (2009). Expression of Erk5 in early stage breast cancer and association with disease free survival identifies this kinase as a potential therapeutic target. PLoS One.

[17] Weldon CB, Scandurro AB, Rolfe KW, Clayton JL, Elliott S, Butler NN (2002). Identification of mitogen-activated protein kinase kinase as a chemoresistant pathway in MCF-7 cells by using gene expression microarray. Surgery.

[18] Esparis-Ogando A, Diaz-Rodriguez E, Montero JC, Yuste L, Crespo P, Pandiella A (2002). Erk5 participates in neuregulin signal transduction and is constitutively active in breast cancer cells overexpressing ErbB2. Mol Cell Biol.

[19] Mehta PB, Jenkins BL, McCarthy L, Thilak L, Robson CN, Neal DE (2003). MEK5 overexpression is associated with metastatic prostate cancer, and stimulates proliferation, MMP-9 expression and invasion. Oncogene.

[20] Zen K, Yasui K, Nakajima T, Zen Y, Zen K, Gen Y (2009). ERK5 is a target for gene amplification at 17p11 and promotes cell growth in hepatocellular carcinoma by regulating mitotic entry. Genes Chromosomes Cancer.

[21] Yang Q, Deng X, Lu B, Cameron M, Fearns C, Patricelli MP (2010). Pharmacological inhibition of BMK1 suppresses tumor growth through promyelocytic leukemia protein. Cancer Cell.

[22] Kato Y, Kravchenko VV, Tapping RI, Han J, Ulevitch RJ, Lee JD (1997). BMK1/ERK5 regulates serum-induced early gene expression through transcription factor MEF2C. EMBO J.

[23] Arias-González L, Moreno-Gimeno I, del Campo AR, Serrano-Oviedo L, Valero ML, Esparís-Ogando A (2013). ERK5/BMK1 is a novel target of the tumor suppressor VHL: implication in clear cell renal carcinoma. Neoplasia.

[24] Buschbeck M, Ullrich A (2005). The unique C-terminal tail of the mitogen-activated protein kinase ERK5 regulates its activation and nuclear shuttling. J Biol Chem.

[25] Rooney M, Devarakonda S, Govindan R (2013). Genomics of squamous cell lung cancer. Oncologist.

[26] Prima V, Hunger SP (2007). Cooperative transformation by MEF2D/DAZAP1 and DAZAP1/MEF2D fusion proteins generated by the variant t(1;19) in acute lymphoblastic leukemia. Leukemia.

