# Supplementary figures and images for: Identification and validation of dysregulated MAPK7 (ERK5) as a novel oncogenic target in squamous cell lung and esophageal carcinoma
Source: BMC Cancer. 2015 Jun 4;15:454. doi: 10.1186/s12885-015-1455-y (PMC4453990; doi:10.1186/s12885-015-1455-y)

## Slide 1
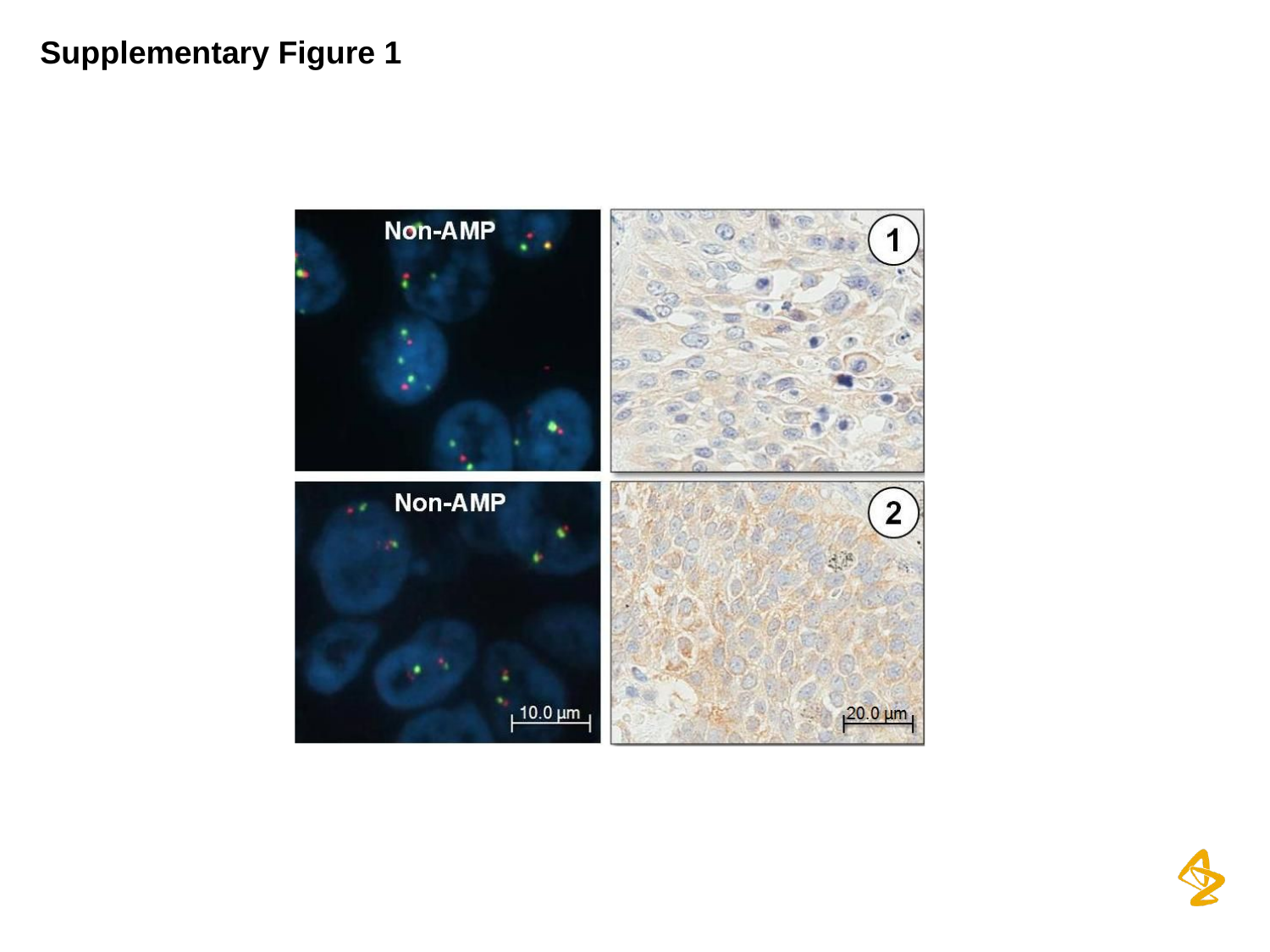

Supplementary Figure 1

Supplement: Additional file 1: Figure S1. — Representative FISH and IHC staining images of NSCLC tissue samples. Circled numbers within the IHC images refer to ‘IHC 1+’ and ‘IHC 2+’ staining intensities. [file 12885_2015_1455_MOESM1_ESM.pptx]

## Slide 1
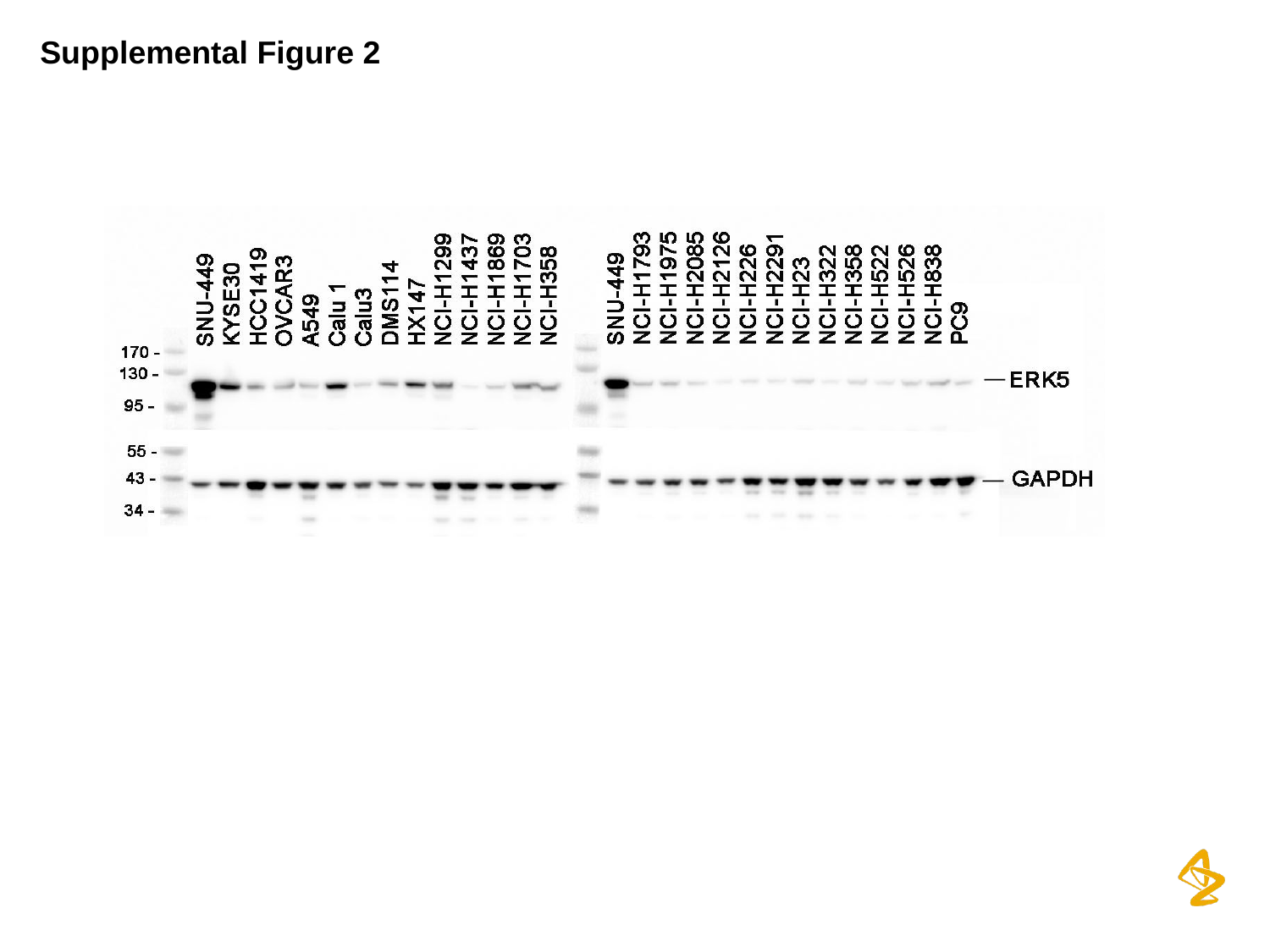

Supplemental Figure 2

Supplement: Additional file 3: Figure S2. — Western blot analysis of tumor cell lines for Erk5 protein. Cell lysates were prepared and analysed for detection of total Erk5 and GAPDH as described in ‘materials and methods’. [file 12885_2015_1455_MOESM3_ESM.pptx]
